# Dysregulation of cadherins in the intercalated disc of the spontaneously hypertensive stroke-prone rat

**DOI:** 10.1016/j.yjmcc.2010.01.017

**Published:** 2010-06

**Authors:** Margaret Anne Craig, Martin W. McBride, Godfrey Smith, Sarah J. George, Andrew Baker

**Affiliations:** BHF Glasgow Cardiovascular Research Centre, University of Glasgow, 126 University place, Glasgow, G12 8TA, UK

**Keywords:** E-cadherin, β-catenin, Cardiac hypertrophy, Intercalated disc

## Abstract

The structural integrity of cardiac cells is maintained by the Ca^2+^-dependent homophilic cell–cell adhesion of cadherins. N-cadherin is responsible for this adhesion under normal physiological conditions. The role of cadherins in adverse cardiac pathology is less clear. We studied the hearts of the stroke-prone spontaneously hypertensive (SHRSP) rat as a genetic model of cardiac hypertrophy and compared them to Wistar–Kyoto control animals. Western blotting of protein homogenates from 12-week old SHRSP animals indicated that similar levels of β, γ-, and α-catenin and T, N and R-cadherin were expressed in the control and SHRSP animals. However, dramatically higher levels of E-cadherin were detected in SHRSP animals compared to controls at 6, 12 and 18 weeks of age. This was confirmed by quantitative Taqman PCR and immunohistochemistry. E-cadherin was located at the intercalated disc of the myocytes in co-localisation with connexin 43. Adenoviral overexpression of E-cadherin in rat H9c2 cells and primary rabbit myocytes resulted in a significant reduction in myocyte cell diameter and breadth. E-cadherin overexpression resulted in re-localisation of β-catenin to the cell surface particularly to cell–cell junctions. Subsequent immunohistochemistry of the hearts of WKY and SHRSP animals also revealed increased levels of β-catenin in the intercalated disc in the SHRSP compared to WKY. Therefore, remodelling of the intercalated disc in the hearts of SHRSP animals may contribute to the altered function observed in these animals.

## 1 Introduction

The cadherins are a super-family of transmembrane glycoproteinsthat mediate homophilic, Ca^2+^-dependent cell–cell adhesion [1–3]. The cadherin mediated cell–cell adhesion provides strong intercellular bonds, which are dependent upon the association of the cadherin carboxyterminal cytoplasmic domain to the central region of β- or γ-catenin and p120 [4–6]. β- and γ-catenin bind α-catenin, in turn promote the anchorage of the complex to the actin cytoskeleton. Free (non-cadherin associated) β-catenin associates in another complex, containing the proteins adenomatous polyposis coli (APC), axin, and glycogen synthase 3β (GSK3β), which targets β-catenin for degradation. Wnt signalling initiated by the binding of Wnt to the Frizzled receptors, antagonises this APC–axin–GSK3β complex, leading to an increase in the pool of free cytoplasmic β-catenin resulting in catenin translocation to the nucleus. Subsequently, β-catenin, in association with T cell factor (TCF), regulates transcriptional activity of numerous target genes, including the cell cycle genes cyclin D1, p21, transcription factors such as c-myc, c-jun and Sox9 and extracellular matrix regulatory genes such as fibronectin, MT1-MMP and MMP-7 [7–10].

The members of the cadherin family have distinct spatial and temporal patterns of expression during embryonic development and in the adult. E-cadherin is expressed in most epithelia throughout the body, while N-cadherin expression is more restricted to specific cell types such as muscle and neurones [2,11]. The function of different cadherin subtypes also depends on the cellular context. In support of this, a switch in cadherin expression from E- to N-cadherin occurs in many cancer types, leading to alterations in cell behaviour [12]. Although it is established that N-cadherin and E-cadherin are crucial for cardiogenesis [13,14], to date, few studies have addressed the expression and role of these cadherins in adult heart tissue. A study by Kostetskii et al, illustrated that deletion of N-cadherin in the mouse heart leads to dilated cardiomyopathy and impaired cardiac function due to loss of N-cadherin-mediated anchorage of myofibrils at the plasma membrane [15]. This study highlights the absolute requirement for N-cadherin in the heart.

The SHRSP is a genetically bred model of hypertension with a high incidence of cerebrovascular events and cardiac remodelling [16,17]. Cardiac hypertrophy is an adaptive response of the heart to increased workload. However sustained cardiac hypertrophy may ultimately lead to heart failure. The transition from compensated hypertrophy to failure of the myocardium involves a complex of events; myocyte growth or hypertrophy, changes in myocyte phenotype resulting from re-expression of foetal gene programs and alterations in the expression or function or both, of proteins involved in excitation–contraction (E–C) coupling and contraction and changes in the extracellular matrix. Together these events result in changes in myocardial structure (e.g. increased myocardial mass, chamber dilatation) and function, often leading to further pump dysfunction and haemodynamic overload [18]. Based on these observations we hypothesised that cadherins may be dysregulated in the remodelling associated with the hearts of the SHRSP model and any alteration in expression may contribute to the alteration in the mechanical and electrical activity observed in these hearts.

## 2 Methods

### 2.1 Animals

Inbred colonies of SHRSP and Wistar–Kyoto (WKY) rats were maintained at the University of Glasgow as described previously [19]. All rats were housed under controlled conditions of temperature (21 °C) and light and maintained on normal rat chow (rat and mouse No. 1 maintenance diet, Special Diet Services, Witham, Essex, UK) and water freely available. These animal studies were approved by the Home Office according to regulations regarding experiments with animals in the UK.

### 2.2 Evaluation of cadherin expression in the myocardium of SHRSP and WKY rats

6, 12 and 18-week old SHRSP animals and WKY controls were euthanized and hearts removed. Tissue was divided and processed for protein homogenates, RNA extraction and immunohistochemistry. For western blotting, proteins were electrophoresed and transferred onto Hybond-P membrane (Amersham Bioscience, Buckingham, U.K.), and blocked with 10% (wt/vol) skimmed milk powder in TBS-T (150 mM NaCl, 50 mM Tris, 0.1% (v/v) Tween-20). The membrane was incubated for 1 h at 37 °C with the following antibodies, N-cadherin, E-cadherin, R-cadherin, T-cadherin, β-catenin, γ-catenin, α-catenin and GAPDH as the loading control (BD Biosciences, Oxford, U.K.). Proteins were visualised using an ECL detection system (Amersham Biosciences, U.K.). For quantification of mRNA levels, quantitative Real-Time PCR was used. Heart RNA was extracted from 12-week old rats using RNeasy kits (Qiagen, West Sussex, U.K.), treated with DNase Free (Ambion, Warrington, U.K.), and quantified using Nanodrop (Labtech, East Sussex, U.K.). Normalisation was confirmed by performing real-time PCR (Taqman, Applied Biosystems, California, U.S.A.) of Actβ (β-actin; Applied Biosystems) with comparable threshold cycles. Taqman probe for E-cadherin (Rn00580109_m1-labelled FAM) were multiplexed with Actβ (4352340E-labelled VIC). Expression of E-cadherin relative to Actβ was derived using the comparative (2^−∆∆ct^) method and is shown as fold change [20]. PCR primers were designed to exon 1 and 16 around SNP1 and SNP2 identified on the Ensembl web site using BN genome sequence as a template. PCR products were prepared with the Agencourt AMPure PCR Purification system (Agencourt Bioscience, Buckinghamshire, U.K.) and sequenced with BigDye v3.1 fluorescent nucleotides (Applied Biosystems). Sequencing reactions were purified with the Agencourt CleanSEQ Sequencing Reaction Clean-Up system and run on the ABI 3730 using polymer 7 and DNA sequence analysed with SeqScape version 2.5 (Applied Biosystems). For immunohistochemistry, formalin-fixed paraffin-embedded tissue sections (6 µm) were sequentially deparaffinised and rehydrated through an alcohol gradient. Tissue sections were incubated with primary anti-E-cadherin, N-cadherin and β-catenin antibodies or matched mouse IgG non-immune control (Dako, Denmark) followed by detection with biotinylated universal secondary antibody (1/200), ABC Kit and standard diaminobenzidine staining. Sections were counterstained with haematoxylin. Serial sections were also stained with a marker for the intercalated disc (connexin 43, Sigma) to aid localisation of β-catenin and E-cadherin.

### 2.3 Construction and propagation of Ad vectors

Adenovirus to overexpress E-cadherin was a gift from Dr D. Jean, University of Sherbrooke, Quebec [21]. Positive plaques were amplified and tested for E-cadherin expression by Western blot analysis. Plaques expressing E-cadherin were amplified and the titer of virus stocks was determined by plaque assay on 293T cells [22].

### 2.4 *In vitro* assessment of myocyte structure

New Zealand White rabbits (2–2.5 kg) were euthanized by administration of an intravenous injection of 500 IU heparin together with an overdose of sodium pentobarbitone (100 mg/kg). Hearts were removed and perfused retrogradely (25 ml/min, 37 °C) with a nominally Ca^2+^ free Krebs–Henseleit solution supplemented with 0.6 mg/ml collagenase and 0.1 mg/ml protease for 6 min. Perfused tissue was finely dissected and filtered through gauze to give a cardiomyocyte cell suspension. To assess the effects of E-cadherin overexpression on function and structure of single cardiomyocytes, Primary rabbit myocytes or H9c2 (a myogenic cell-line derived from embryonic rat ventricle) cells were counted, and adenoviral infection was performed during plating of the myocytes at 3 × 10^4^ rod-shaped cells into Petri dishes with either Ad:E-cadherin or Ad:*Lac*Z with an indicated multiplicity of infection (MOI). Myocytes were cultured in supplemented M199 medium (Sigma) for 48 h. The lysate of cells transfected with Ad–E-cadherin were subjected to western immunoblotting as before. Infection of H9c2 and isolated rabbit myocytes with increasing doses of Ad:E-cadherin allowed for the effects of dose response on hypertrophy to be assessed by the calculation of cell volume. Cell length was measured 48 h post-transfection. Samples of the cell suspension were placed on a glass slide and measured with a graticule in the eye-piece of the microscope and cell volume calculated (*n* = 50 per group).

### 2.5 Immunocytochemistry of H9c2 cells infected with the E-cadherin adenovirus

H9c2 cells were infected with either the Ad:E-cadherin or Ad:*Lac*Z at an MOI of 30. 48 h later the cells were washed in PBS and fixed with 10% formaldehyde. Fixed cells were incubated with primary anti-E-cadherin, N-cadherin and β-catenin antibodies or matched mouse IgG non-immune control (Dako, Denmark) followed by detection with FITC conjugated universal secondary antibody. The cells were mounted with vectashield with DAPI and visualised by fluorescent microscopy.

### 2.6 Immunoprecipitation of E-cadherin with β-catenin

H9c2 cells were grown in a monolayer and infected with either Ad:E-cadherin or Ad:*Lac*Z at an MOI of 30. Following 48 h, the cells were harvested in RIPA buffer. Equal protein concentrations of the lysed cells were incubated with either β-catenin-agarose conjugated antibody or IgG-agarose conjugated control at 4 °C overnight. A separate sample of Ad-transduced cells was incubated overnight without antibody and was used to quantify the total level of E-cadherin. The cell lysate was then washed with RIPA buffer and loading buffer added. The cell sample was then probed for E-cadherin as described before.

### 2.7 Statistical analysis

Comparisons were made using the student's *t*-test. Statistical analysis was performed in Prism version 4.0 (Graph Pad Software, CA, USA). For all tests, *P* < 0.05 is considered statistically significant. The results represent mean values and SEM of the data.

## 3 Results

**Fig. 1:**
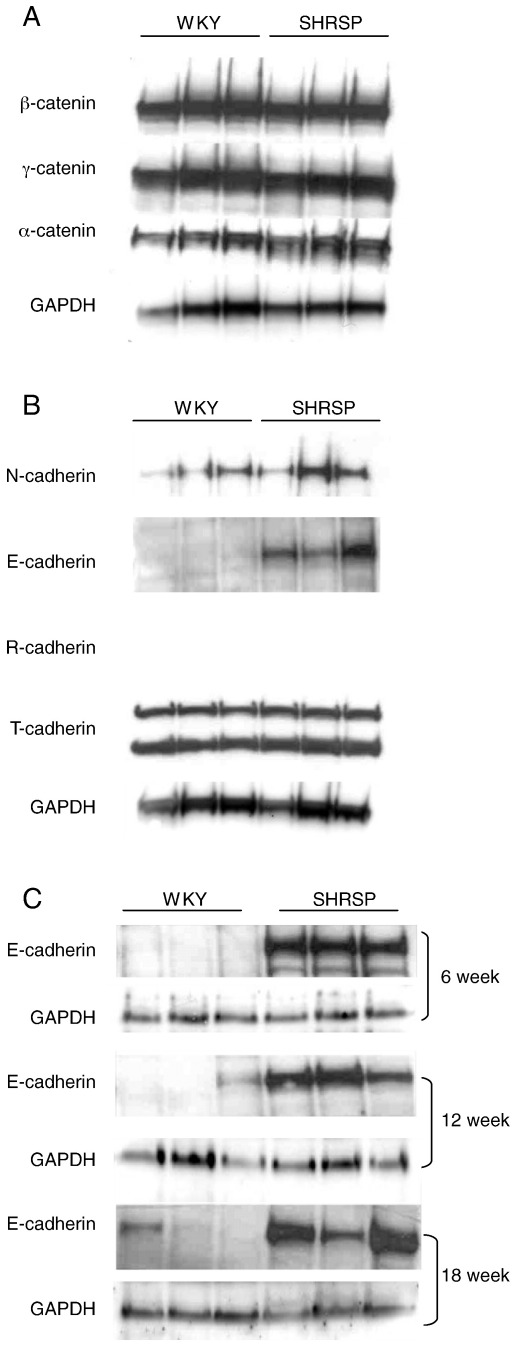
Cadherin and catenin expression in the SHRSP and WKY rat hearts. Western blot analysis of heart extracts from 12-week old SHRSP and WKY animals (shown as 3 of each, *n* = 9). (A) Western blot analysis from heart protein homogenates prepared from animals 12 weeks of age indicated that the levels of β-, γ- and α-catenin were all unchanged between the control and the SHRSP animals. (B) Levels of T-, N- and R-cadherin also remained unchanged, however we observed a marked up-regulated level of E-cadherin from low levels in the WKY to readily detectable levels in the SHRSP. (C) Further analysis of heart homogenates from 6, 12 and 18 week old animals showed these cadherin levels were up-regulated from as early as 6 weeks of age through to 18 weeks.

**Fig. 2:**
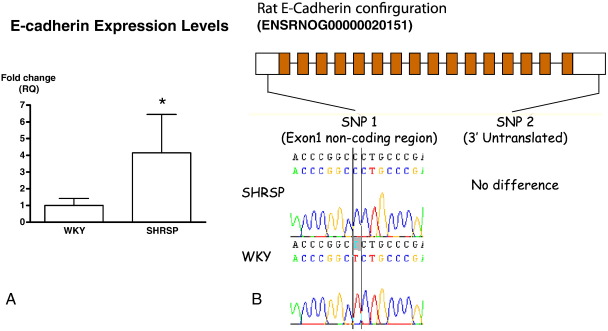
Altered E-cadherin expression. (A) Taqman gene expression analysis of RNA extracted from the hearts of 12-week old SHRSP and WKY animals. Data shows a significant increase in expression of E-cadherin in the SHRSP group and is shown as relative fold change (*n* = 6). (B) Genome organisation of E-cadherin. Sequence analysis identified a nucleotide change for SNP1 located in the non-coding region of exon 1.

We first sought to assess whether cadherin/catenin expression levels were modulated in the SHRSP compared to its normotensive control strain WKY. Western blot analysis from heart protein homogenates prepared from animals 12 weeks of age indicated that the levels of β-, γ- and α-catenin were all similar between the control and the SHRSP animals, as were levels of T-, N- and R-cadherin (Fig. 1(A)). However, we observed a marked up-regulation of E-cadherin from low levels in the WKY to readily detectable levels in the SHRSP (Fig. 1(B)). Further analysis of heart homogenates from 6, 12 and 18 week old animals showed E-cadherin levels were up-regulated from as early as 6 weeks of age and still high at 18 weeks (Fig. 1(C)). Taqman quantitative RT-PCR gene expression analysis confirmed significantly elevated E-cadherin levels in the SHRSP animals compared to WKY controls (Fig. 2(A)). The data suggests that there is a 4.14-fold increase in the level of E-cadherin (WKY mean ∆Ct = 12.86 ± 0.50, SHRSP ∆Ct = 10.81 ± 0.64, *p* = 0.03). This suggests a transcriptional control mechanism that leads to E-cadherin overexpression in the SHRSP rather than post-transcriptional control. Interestingly, following amplification of 5′ and 3′ regions of the rat E-cadherin gene we observed a single nucleotide polymorphism in the 5′ non-coding region (exon 1) between the SHRSP and WKY (Fig. 2(B)).

**Fig. 3:**
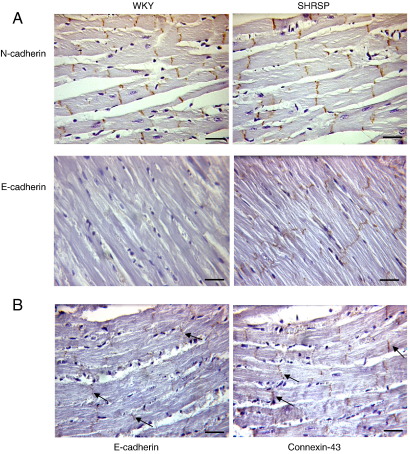
Histological analysis of hearts from the SHRSP and WKY. Histological analysis was carried out on the hearts of 12-week old WKY and SHRSP animals. (A) There appeared to be no difference in levels of N-cadherin between the SHRSP animals and the WKY controls. Immunohistochemistry of tissue sections for E-cadherin demonstrated up-regulated levels of E-cadherin in the SHRSP compared to the WKY control (scale = 25 µm). (B) Immunohistochemical staining of SHRSP hearts showing localisation of E-cadherin to the intercalated disc similar to the adherens junction marker connexin 43 (scale = 25 µm).

We next evaluated the localisation of E- and N-cadherin in heart sections from animals at 6, 12, and 18 weeks of age using immunohistochemistry. N-cadherin was located in the intercalated disc as expected. The levels of N-cadherin were similar in WKY and SHRSP (Fig. 3(A)). In contrast, the levels of E-cadherin were higher in the SHRSP animal compared to the WKY (Fig. 3(A)), confirming the western blotting and real-time PCR results. The E-cadherin staining was located in the intercalated disc and was found to be co-localised with connexin 43 (Fig. 3(B)).

**Fig. 4:**
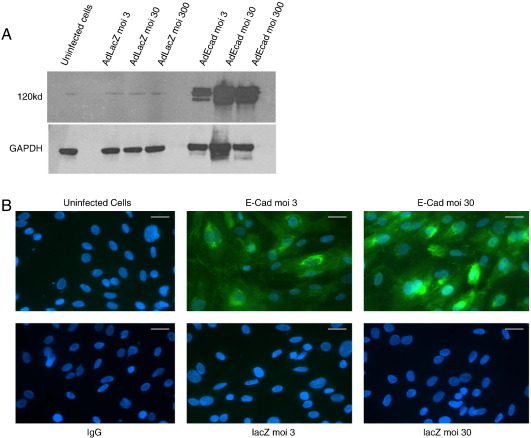
Adenovirus mediated *in vitro* overexpression of E-cadherin. Isolated single adult rabbit cells or H9c2 cells were infected with either Ad:E-cadherin or Ad:*LacZ*. (A) Western blot from extracts of H9c2 cells infected with E-cadherin showing increasing levels of E-cadherin from differing multiplicities of infection and the GAPDH loading control (B) Immunocytochemical levels of E-cadherin in H9c2 cells infected with either Ad:E-cadherin or Ad:*LacZ*. Successful infection of E-cadherin into the H9c2 cell-line can be observed at low and high levels of infection (scale = 25 µm).

**Fig. 5:**
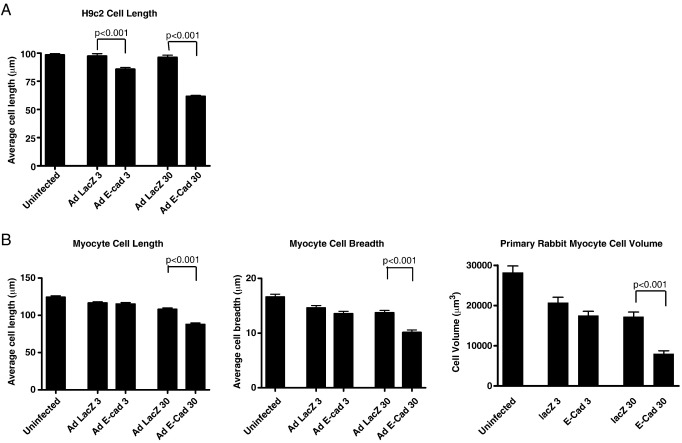
Effect of E-cadherin overexpression on cell dimensions. Hearts from adult rabbits were excised and single cells enzymatically isolated using a Langendorff perfusion system. Isolated single adult rabbit cells and H9c2 cells were infected with either Ad:E-cadherin or Ad:*LacZ* Cell dimensions were measured using a graticule in the eye-piece of a microscope. (A) Transduction of H9c2 cells with AD:E-cadherin resulted in significantly decreased cell diameter (*n* = 50/group). (B) Infection of rabbit myocytes with Ad:E-cadherin also resulted in cells with a significantly decreased cell length and width resulting in an overall decrease in cell volume (*n* = 50/group).

We next sought to investigate the potential effect of E-cadherin dysregulation in the heart by using an *in vitro* model system. We used rat H9c2 cells, an established cell model system for studies on myocyte hypertrophy [23,24] as well as primary rabbit myocytes [25]. We first used an adenovirus to overexpress E-cadherin in H9c2 cultures. Efficient transduction and ectopic E-cadherin expression was observed by western blot analysis and immunocytochemistry (Fig. 4). E-cadherin overexpression resulted in a significant decrease in H9c2 cell diameter (Fig. 5(A)). Overexpression of E-cadherin in the primary rabbit myocytes also affected cell shape and size; a significant reduction of both cell length and width resulting in an overall reduction of cell volume was detected (Fig. 5(B)).

**Fig. 6:**
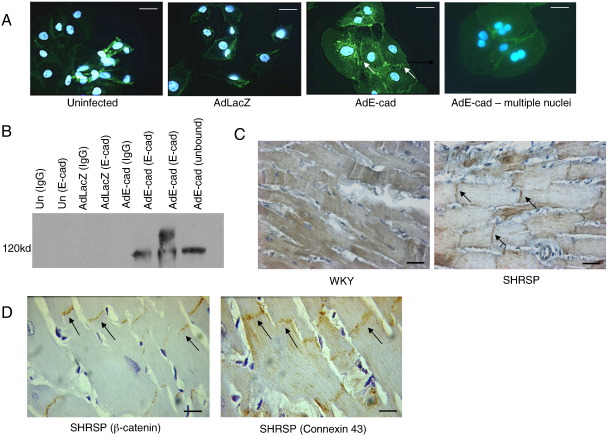
Localisation of β-catenin following E-cadherin overexpression. H9c2 cells were infected with either Ad:E-cadherin or Ad:*LacZ* and levels and localisation of N-cadherin and β-catenin were analysed. (A) Immunocytochemistry of H9c2 cells overexpressing E-cadherin showing up-regulation of β-catenin both in the cytoplasm but also markedly at the cell-to-cell junctions (indicated by the arrows). Many of the infected cells also contained two or more nuclei (B) H9c2 cells infected with either Ad:E-cadherin or Ad:*LacZ*. Cells were lysed in RIPA buffer and undergone immunoprecipitation with either β-catenin or IgG control antibody. Western blot showing that much of the E-cadherin co-precipitates and therefore co-localises with β-catenin. (C) Immunohistochemical localisation of β-catenin in the hearts of 12-week old SHRSP and WKY controls. There appears to be selective localisation of β-catenin at the intercalated disc of the cell membrane in the SHRSP hearts (indicated by the arrows, scale = 10 µm). (D) Immunohistochemical staining of WKY and SHRSP hearts showing localisation of β-catenin to the intercalated disc similar to the adherens junction marker connexin 43 (scale = 10 µm).

To ascertain the effect of E-cadherin overexpression on β-catenin levels and localisation we assayed control and E-cadherin adenovirus-infected cells 48 h post-transduction. Uninfected cells showed low levels of β-catenin staining in the cytoplasm. Increased expression of E-cadherin resulted in selective localisation of β-catenin to the cell surface especially in regions of cell–cell contact (Fig. 6(A)). Increased expression of E-cadherin also resulted in greater than 50% of cells having two or more nuclei. To assess whether β-catenin associated with E-cadherin we performed co-immunoprecipitation experiments. Western blotting for E-cadherin demonstrated interaction with β-catenin however, the results indicated that not all of the E-cadherin was bound to β-catenin (Fig. 6(B)). Finally, we assessed the localisation of β-catenin *in vivo* in the hearts of SHRSP and WKY animals. β-catenin appeared to be selective to the intercalated disc in SHRSP hearts (Figs. 6(C) and (D)).

## 4 Discussion

The structural and electrical integrity of ventricle muscle is maintained by the end-to-end connection between myocytes termed the intercalated disc. In the adult heart this consists of three junctional complexes; zona adherens, desmosomes and gap junctions [26]. The reorganisation of homophilic cell–cell adhesion is mainly regulated by the cadherin/catenin system, which is differentially modulated to sustain cell structural rather than signalling needs. N-cadherin, which is normally expressed in the adult heart is required for maintenance of the structural integrity and myofibril continuity across the plasma membrane [27,28]. In the SHRSP model, levels of N-cadherin appeared to be relatively unaltered compared to controls. However E-cadherin was virtually absent in normotensive WKY animals consistent with observations in the hearts of normal mice [29], was up-regulated in the hearts of SHRSP rats, as assessed by immunohistochemistry, western blot and Taqman PCR analysis at 6, 12 and 18 weeks of age. However, the consequences of this alteration in cadherin type in the myocardium in the SHRSP is unclear. A study in which E-cadherin was substituted for N-cadherin in the heart of N-cadherin null mice, caused premature death occurred prior to intercalated disc formation and pathological features similar to that found in human end stage heart disease [30]. Of note, E-cadherin overexpression led to DNA synthesis and binucleated myocytes, associated with increased cyclin D1 expression. Our *in vitro* studies also showed that overexpression of E-cadherin into the H9c2 cell-line resulted in many of the cells having two or more nuclei.

The detected increase in E-cadherin in SHRSP rat hearts may be a result of altered transcriptional activity. Based on the published sequence of the rat genome we have amplified two regions of the rat E-cadherin gene by PCR from genomic DNA and assessed known single nucleotide polymorphisms (SNPs). Of the two known SNPs, the SNP positioned in the 5′ non-coding region of exon 1 was different between WKY and SHRSP animals. This may have a further impact on the effect of dysregulated E-cadherin expression in the SHRSP.

Although levels of the other cadherins and catenins appeared to be unaltered, distribution of β-catenin was different. In E-cadherin overexpressing cells, β-catenin was detected at cell–cell junctions and in association with E-cadherin, which may restrict WNT/β-catenin signalling. In heart development, β-catenin plays a biphasic role in cardiomyocyte differentiation; initially in committing mesenchymal cells to the cardiac lineage, while at later stages a down-regulation is required for differentiation [31]. β-catenin levels are therefore relatively high in the embryonic heart compared to the adult heart. In hypertrophic cardiomyopathic hearts accumulation of β-catenin particularly in the intercalated discs occurs as a result of Wnt expression, decrease in GSK-3β and different localization of APC [32].

The accumulation of β-catenin in intercalated discs is thought to play a role in determining the previously described plasmalemma rigidification in δ-SG null cardiomyocytes [32]. This mechanism could contribute to increased myocardial wall stiffness and left ventricular end-diastolic pressure as well as to modify intercellular electric impedance in hypertrophic cardiomyopathic hamsters and men. In human hypertrophic cardiomyopathy there is also found to be a remarkable accumulation of N-cadherin and β-catenin resulting in enlarged and disorganised intercalated discs [32]. Overexpression of E-cadherin in isolated cardiomyocytes and the H9c2 cell-line resulted in a significant decrease in cell volume and more rounded cells. The cell-to-cell adhesion that is associated with cadherins, requires re-arrangement of the actin cytoskeleton. Loss of N-cadherin results in the disassembly of the intercalated disc structure, including adherens junctions and desmosomes. Decreased sarcomere length and increased Z-line thickness were observed in the mutant hearts consistent with loss of muscle tension because N-cadherin was no longer able to anchor myofibrils to the plasma membrane [15]. We propose that introduction of E-cadherin into these cell types may also result in disorganised myofibril structure. Conversely, it may result in a stiffening of myofibril structure and may contribute to the increased stiffness observed in cardiac hypertrophy.

We have shown for the first time that increased E-cadherin and β-catenin are dysregulated in the hearts of the SHRSP and therefore may contribute in the pathology of cardiac disease. This association of aberrant pathology with E-cadherin/β-catenin has however been observed in animal models of cancer such as colon cancer [33]. Increased expression of E-cadherin in the crypts of the colon results in the up-regulation of APC protein, some of which enters the nucleus. There it makes the cells susceptible to the eventual apoptotic balancing by stopping survivin expression and the β-catenin–TCF-4 complex from driving further cell cycling by releasing β-catenin from the ultimate proteasomal destruction. Cytoplasmic β-catenin is then prevented from returning to the nucleus by either being intercepted and destroyed by the APC–axin–GSK-3β complexes or locked by the emerging E-cadherin into the membrane adherens junctions, which tie the cell into a sheet of proliferatively shut-down cells. In fact, the segregation of β-catenin in intercalated disc implies that cytoplasmic β-catenin level is lowered and nuclei are deprived of stimuli needed to maintain a physiological gene activity.

To evaluate the effect of E-cadherin on cardiac function, we transfected adult rabbit ventricular myocytes with E-cadherin adenovirus and measured their fractional shortening in response to field stimulation. The cells overexpressing E-cadherin maintained normal function (data not shown). Dependent on the intracellular Ca^2+^ concentration, E-cadherin has been shown to assemble and increase the function of connexin 43 [34].Therefore, re-introduction of the foetal oncogene E-cadherin may be a compensatory mechanism to up-regulate the levels of connexins and therefore aid cardiac function. This mechanism may be further aided by relocation of β-catenin to the intercalated disc since deletion of β-catenin in stress induced cardiac hypertrophy resulted in a 33% reduction in the expression of connexin 43 [35].

Dysregulation of the intercalated disc in the SHRSP model may therefore result in alterations in structure and cell-signalling which may contribute to the aberrant structure and function observed in these animals. Together, these findings may provide an insight into the pathology observed in heart disease.
